# Pharmacokinetic Evidence Supporting Subcutaneous Use of Protein C Concentrate in Patients with Protein C Deficiency

**DOI:** 10.1055/a-2731-5372

**Published:** 2025-11-11

**Authors:** Zhaoyang Li, Inmaculada C. Sorribes, Jennifer Schneider, Adekemi Taylor

**Affiliations:** 1 Takeda Development Center Americas, Inc., Cambridge, Massachusetts, United States; 2 Certara, Inc., Radnor, Pennsylvania, United States

**Keywords:** population pharmacokinetics, protein C concentrate, protein C deficiency, replacement therapy, subcutaneous

## Abstract

**Background:**

Protein C concentrate (Ceprotin®; Baxalta US Inc., a Takeda company, Cambridge, MA; Takeda Manufacturing Austria AG, Vienna, Austria) is approved for intravenous (IV) use in severe congenital protein C deficiency (SCPCD), with pharmacokinetic (PK)-guided dosing. Subcutaneous (SC) administration may reduce treatment burden, especially for pediatric and neonatal patients; however, the use of SC protein C concentrate has so far been empirical, and PK data are required to support dose optimization.

**Objectives:**

This study aimed to characterize the population PK (PopPK) of SC protein C concentrate in patients with SCPCD.

**Methods:**

A PopPK model was developed for SC protein C concentrate, based on a previously developed model for IV administration. Simulations were conducted across eight three-stage dosing scenarios that patterned the IV dosing regimens in the U.S. product label (initial dose [stage 1]: 60–120 IU/kg; subsequent three doses [stage 2]: 60–80 IU/kg every 6 hours; maintenance dose [stage 3]: 45–120 IU/kg every 12 hours). Additional simulations were performed across six one-stage dosing scenarios that were based on dosing reported in clinical practice (50–60 IU/kg every 12 hours, 200–350 IU/kg every 48 hours). Target maximum ( *C*_max_ ) and trough ( *C*_trough_ ) concentration levels used as references were 100 IU/dL and 25 IU/dL, respectively.

**Results:**

The dataset included 86 observations from 13 patients with SCPCD receiving SC protein C concentrate. Model-based simulations predicted that, after the first dose, 6–9% and 5–45% of patients in the three- and one-stage dosing scenarios, respectively, would attain *C*_max_ >100 IU/dL. At steady state, ≥83% of patients were predicted to attain *C*_trough_ >25 IU/dL for all scenarios. In three-stage dosing scenarios, while initial (stage 1 [dose 1]) and subsequent doses (stage 2 [doses 2–4]) determined speed to steady state, exposure at steady state was driven by the maintenance dose (stage 3 [dose 5 onwards]).

**Conclusions:**

The PopPK model was robust and described SC protein C concentrate PK data well. Evidence provided by model-based simulations supports the use of various SC dosing regimens across age groups in acute or prophylactic settings according to the intended protein C activity levels. A high loading dose may be required to rapidly attain target therapeutic concentrations.

## Introduction

Protein C is an endogenous vitamin K-dependent anticoagulant. In healthy term infants, the mean plasma concentration is 40 IU/dL with a lower limit of normal at 25 IU/dL, 1 while in healthy adults the range of plasma concentrations is 65 to 135 IU/dL. 2 Protein C deficiency is a rare but serious disorder that can be congenital or acquired secondary to various conditions that increase the consumption, or decrease the synthesis, of protein C. 1 3 Patients with protein C deficiency are at risk of developing disseminated intravascular coagulation (DIC) or venous thromboembolism. Severe congenital protein C deficiency (SCPCD) is a recessive disorder that results from homozygous or compound heterozygous mutations in the *PROC* gene. In infants, SCPCD leads to purpura fulminans and DIC within hours after birth, and if left untreated, it can lead to multiple organ failure and ultimately death. Even in survivors, blindness and long-term neurological effects are common. 1 4 5

Replacement therapy with intravenous (IV) protein C concentrate purified from human plasma (Ceprotin®; Baxalta US Inc., a Takeda company, Cambridge, MA; Takeda Manufacturing Austria AG, Vienna, Austria) is approved for the management of SCPCD, but not for acquired forms of the condition. 6 7 For acute episodes or short-term prophylaxis of venous thrombosis and purpura fulminans, the U.S. product label recommends an initial dose of 100–120 IU/kg, followed by three doses of 60–80 IU/kg given every 6 hours (Q6h), and maintenance dosing of 45–60 IU/kg given either Q6h or every 12 hours (Q12h). 6 In the European Medicines Agency (EMA) summary of product characteristics (SmPC), the target is a protein C activity of 100% (100 IU/dL) initially, maintained above 25% for the duration of treatment. 7 An initial dose of 60–80 IU/kg is recommended, which can be gradually reduced to Q12h to maintain activity above 25% if the initial response is satisfactory. For long-term prophylaxis, a maintenance dose of 45–60 IU/kg Q12h is advised in both the U.S. product label and the EMA SmPC. 6 7

Although protein C concentrate is approved for IV administration, several articles have reported subcutaneous (SC) administration in patients with SCPCD in clinical practice. 5 8 9 10 11 12 13 14 15 16 17 The EMA SmPC for protein C concentrate notes that, in rare and exceptional cases, SC infusion of 250–350 IU/kg was able to produce therapeutic protein C plasma levels in patients with no IV access. 7 In addition, the International Society on Thrombosis and Haemostasis (ISTH) Scientific and Standardization Committees (Plasma Coagulation Inhibitors, Pediatric/Neonatal Thrombosis and Hemostasis, and Women's Health Issues in Thrombosis and Hemostasis) recommended the use of SC administration of protein C concentrate for acute management in exceptional circumstances when venous access is not feasible, and as the most appropriate long-term prophylaxis to avoid potential central venous access problems. 5 The ISTH recommendations recognize that although SC administration is not licensed, it has been successfully used off-label for more than 25 years, with SC use associated with benefits in the context of long-term management. 5 Other potential benefits of SC administration include patient convenience and reducing the frequency of injections. 18 In an online survey, 19 physicians with experience using protein C concentrate reported that 12 of their treated patients in Europe received long-term prophylaxis via SC administration, and 18 physician respondents from Europe and the U.S. indicated their interest in having SC administration as an approved route of administration for protein C concentrate. The current administration of SC long-term prophylaxis was reported by physicians in Europe alone. 19

The dosing of protein C concentration is guided by pharmacokinetics (PK). The dose should be adjusted based on laboratory assessments for each individual and determined based on the protein C activity in plasma. Therefore, an understanding of the PK of protein C concentrate is crucial. To date, PK data on the SC administration of protein C concentrate in the literature are sparse. Population PK (PopPK) modeling is a powerful tool to help understand PK data at the patient population level and subsequently to guide appropriate dosing regimens, particularly as the treatment of protein C deficiency is pharmacokinetically guided to maintain trough concentrations ( *C*_trough_ ) of >25 IU/dL. 6 7 PopPK modeling allows concentration–time data to be pooled from more than one source to predict the population and individual exposure response in a target population and simultaneously identify the potential patient factors that impact PK data. 20

In this study, we characterized the PK of SC protein C concentrate using a PopPK model that was based on a previously developed model for IV administration and incorporated literature summaries on SC administration. Model-based simulations were then conducted for a range of clinical dosing regimens to assess the effectiveness of each regimen in reaching the target plasma protein C activity at various stages of dosing.

## Methods

### Subcutaneous Pharmacokinetic Dataset

PK data were extracted from six literature summaries describing SC administration of protein C concentrate. 8 9 10 11 12 13 A seventh literature summary was also assessed, 21 but no data from this publication were included in the analysis owing to a lack of information about the time of dose administration and/or PK sampling. Patients were defined as evaluable for PopPK analysis if they had at least one dose of protein C concentrate administered via the SC route and at least one measurable protein C concentration with its associated sampling time and dosing information. It was essential to build the PopPK model with assumptions of missing information necessary for the analysis. The data imputations associated with the literature are described in the Supplementary Materials.

### Population Pharmacokinetic Modeling

The PopPK model for SC protein C concentrate administration was developed as detailed in the Supplementary Materials by leveraging the PopPK model previously developed for IV administration from four prospective studies of SCPCD or severe acquired protein C deficiency (SAPCD, *n* = 58 patients). 22 The model building and finalization process followed the common standard process, which has also been adopted by the U.S. Food and Drug Administration (FDA) and EMA. 20 23

### Simulations

A representative patient population for SC simulation was created from a virtual population using the U.S. Centers for Disease Control National Health and Nutrition Examination Survey database, using PK-Sim software (version 8; Open Systems Pharmacology, Hauptsitz, Germany). 24 25 The simulation dataset included 2,500 virtual patients, with 500 in each age group (neonates [0–27 days old], infants [28 days to <2 years old], children [2 to <12 years old], adolescents [12 to <16 years old], and adults [≥16 years old]). Equal numbers of male and female patients were included in the dataset, and, as disease type was a selected covariate in the final PopPK model, equal numbers of patients within each age group were randomly assigned to either SCPCD or SAPCD. A fixed interindividual variability (IIV) of 10% coefficient of variation was introduced to SC absorption parameters for simulations.

Maximum plasma concentration ( *C*_max_ ) and *C*_trough_ (a term that is interchangeable with minimum concentration in this study) were calculated and summarized over five different 12-hour-long periods of time: 0–12, 12–24, 24–36, 36–48, and 264–276 hours. The first four periods match the initial and subsequent dosing periods, as outlined in the product label, and the last period represents steady state. 6 Simulation scenarios 1 to 8 (three-stage dosing including initial, subsequent, and maintenance dosing that was patterned after the specified IV dosing regimens in the U.S. product label for protein C concentrate) were selected to align with the highest and lowest recommended doses in the product label for IV administration with different combinations of dose levels ( Table 1 ). 6 7 Scenarios 1 to 8 consisted of an initial dose (60 or 120 IU/kg) followed 12 hours later by three subsequent doses (60 or 80 IU/kg Q6h), and a maintenance dose (45 or 120 IU/kg Q12h) starting 12 hours after the final subsequent dose. Six additional scenarios (9–14) were selected to explore one-stage dosing, including higher, less frequent dosing (200–350 IU/kg every 48 hours [Q48h], scenarios 11–14) based on the EMA SmPC 7 and previous case reports ( Table 1 ). 10 21 26 The proportions of patients who reached target *C*_max_ >100 IU/dL and *C*_trough_ >25 IU/dL after the initial dosing period (0–12 hours) and at steady state (264–276 hours) were evaluated.

**Table 1:** Population pharmacokinetic simulation clinical dosing scenarios a using three-stage dosing (initial, subsequent, and maintenance) and one-stage dosing (patterned after the intravenous dosing regimens in the U.S. product label) for subcutaneous administration of protein C concentrate

Scenario	Initial dose (IU/kg)	Subsequent three doses (IU/kg)	Maintenance dose (IU/kg)
Three-stage dosing			
S1 (lowest doses)	60	60 Q6h	45 Q12h
S2	60	60 Q6h	120 Q12h
S3	60	80 Q6h	45 Q12h
S4	60	80 Q6h	120 Q12h
S5	120	60 Q6h	45 Q12h
S6	120	60 Q6h	120 Q12h
S7	120	80 Q6h	45 Q12h
S8 (highest doses)	120	80 Q6h	120 Q12h
One-stage dosing b			
S9	–	–	50 Q12h c
S10	–	–	60 Q12h
S11	–	–	200 Q48h
S12	–	–	250 Q48h
S13	–	–	300 Q48h
S14	–	–	350 Q48h

Abbreviations: Q6h, every 6 hours; Q12h, every 12 hours; Q48h, every 48 hours; S, scenario.

a In scenarios 1 to 8, the second dose was administered 12 hours after the initial dose, and the first maintenance dose was administered 12 hours after the third subsequent dose.

b In scenarios 9 to 14, a constant dosage was administered throughout the treatment period.

c This dose is equivalent to 200 IU/kg Q48h.

## Results

### Patient Demographics

PK data were extracted from 13 symptomatic patients receiving SC protein C concentrate, whose demographics are presented in Table 2 . The median patient age was 0.169 years (range 0.003–18.0), and the mean body weight was 10.3 kg (standard deviation [SD] 15.6). All patients had SCPCD; approximately half of the patients were female (53.8%), and the mean protein C level at baseline was 22.8 IU/dL (SD 22.1).

**Table 2:** Summary of baseline demographics of patients included in the population pharmacokinetic analyses

Parameter	Boey et al (2016) 8 ( *n* = 1)	de Kort et al (2011) 9 ( *n* = 1)	Minford et al (2014) 10 a ( *n* = 5)	Olivieri et al (2009) 11 ( *n* = 1)	Piccini et al (2014) 12 ( *n* = 1)	Sanz-Rodriguez et al (1999) 13 b ( *n* = 4)	Overall ( *n* = 13)
Age, years							
Mean (SD)	18.0 (–)	0.005 (–)	1.97 (1.65)	–	0.003 (–)	0.003 (–)	2.32 (5.13)
Median (range)	18.0 (–)	0.005 (–)	1.75 (0.33–4.50)	–	0.003 (–)	0.003 (0.003–0.003)	0.169 (0.003–18.0)
Sex, *n* (%)							
Male	0	1 (100)	5 (100)	0	0	0	6 (46.2)
Female	1 (100)	0	0	1 (100)	1 (100)	4 (100)	7 (53.8)
Body weight, kg							
mean (SD)	60.0 (–)	3.20 (–)	11.2 (3.27)	1.64 (–)	2.70 (–)	2.70 (–)	10.3 (15.6)
Indication							
Congenital	1 (100)	1 (100)	5 (100)	1 (100)	1 (100)	4 (100)	13 (100)
Acquired	0	0	0	0	0	0	0
Protein C level (IU/dL)							
mean (SD)	2.5 (–)	2.0 (–)	24.7 (14.3)	73.9 (–)	20.0 (–)	18.8 (22.4)	22.8 (22.1)

Abbreviation: SD, standard deviation.

a This publication reports a series of 14 patients who received treatment with subcutaneous protein C concentrate. Only five patients had serial pharmacokinetic samples and were eligible for inclusion in this analysis.

b This publication was a case study of one patient with severe congenital protein C deficiency who received treatment with subcutaneous protein C concentrate. As the patient received four diﬀerent doses of protein C concentrate, the sample size is reported as *n* = 4.

### Population Pharmacokinetic Modeling

**Fig. 1:**
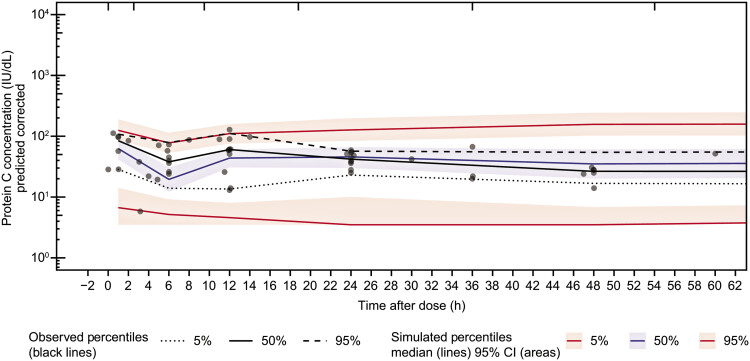
Prediction-corrected visual predicted check plots for the final PopPK model of the SC administration of protein C concentrate. CI, confidence interval; PopPK, population pharmacokinetic; SC, subcutaneous.

In total, 86 observations from the 13 symptomatic patients were included in the analysis. Final model PK parameters for SC protein C concentrate are shown in Table 3 . After testing different models of absorption, a constant (i.e., zero-order) rate of administration into the SC depot compartment, followed by first-order absorption into the central compartment, was found to be the best absorption structure ( Supplementary Fig. S1 ). Absorption parameters were estimated with good precision, with relative standard errors of approximately 20% or lower. The duration of administration was estimated as 4.49 hours, the first-order absorption rate constant was estimated as 0.0379 1/h, and bioavailability was estimated as 79% ( Table 3 ). The performance of the model was confirmed by the goodness-of-fit plots, which showed good agreement between observed versus individual-predicted concentrations of protein C concentrate. The observed versus population-predicted concentrations did not follow the line of unity ( Supplementary Fig. S2 ); however, the prediction-corrected visual predictive check was generally acceptable, as it described the data well in terms of the median trend ( Fig. 1 ).

**Table 3:** Final population pharmacokinetic parameters for subcutaneous protein C concentrate

Parameter	Population estimate	Standard error	Percentage relative standard error	Bootstrap median (2.5th, 97.5th percentiles)
Clearance (dL/h)	7.14	Fixed values a		
Volume of distribution (dL)	60.7			
Effect of age on volume of distribution	−0.112			
Rate of endogenous protein C production (IU/h)	203			
Effect of age on the rate of endogenous protein C production	0.589			
Proportional error CV	0.185			
Additive error SD (IU/dL)	2.5			
Effect of SCPCD indication on clearance	−0.575			
Effect of SCPCD indication on rate of endogenous protein C production	−0.967			
IIV variance on clearance	0.0992			
IIV variance on volume of distribution	0.0505			
IIV variance on the rate of endogenous protein C production	0.103			
First-order absorption rate constant (1/h)	0.0379	0.00161	4.26	0.04 (0.0284, 0.0867)
Bioavailability	0.79	0.0424	5.37	0.764 (0.511, 0.886)
Duration of administration (h) b	4.49	0.91	20.3	4.94 (3.34, 6.37)

Abbreviations: CV, coefficient of variation; IIV, interindividual variability; SC, subcutaneous; SCPCD, severe congenital protein C deficiency; SD, standard deviation.

Allometric scaling was applied to clearance and volume of distribution with fixed exponents of 0.75 and 1, respectively, and a reference body weight of 70 kg. The effects of age are reported as the exponent of the power model, and the effects of SCPCD indication are reported as the fractional change relative to the severe acquired protein C deficiency (SAPCD) indication. The reference age was 4.1 years, and the reference indication was SAPCD. The approximate estimated IIV CVs were 31.5%, 22.5%, and 32.1% for clearance, volume of distribution, and rate of endogenous protein C production, respectively. The condition number for the model was 2.4. In total, 99.4% of bootstrap runs minimized successfully.

a Fixed values were derived from the intravenous population pharmacokinetic model.

b Value was not provided in the literature and was therefore estimated.

### Model-Based Simulations

**Fig. 2:**
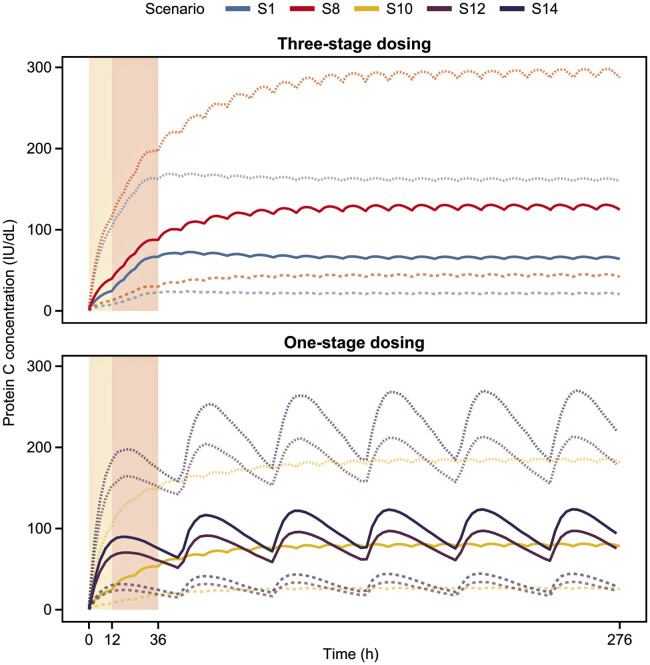
Simulated protein C concentrate concentration–time profiles showing representative three-stage and one-stage dosing scenarios (S1, S8, S10, S12, and S14) up to steady state. The top panel shows simulations for the lowest (S1 [initial: 60 IU/kg; subsequent: 60 IU/kg Q6h; maintenance: 45 IU/kg Q12h]) and highest (S8 [initial: 120 IU/kg; subsequent: 80 IU/kg Q6h; maintenance: 120 IU/kg Q12h]) three-stage dosing scenarios. The bottom panel shows simulations for low (S10 [60 IU/kg Q12h]), medium (S12 [250 IU/kg Q48h]), and high (S14 [350 IU/kg Q48h]) one-stage dosing scenarios. Details of the dosing regimens in each scenario are shown in Table 1 . Median values are shown in solid lines, 5th percentiles are shown in dashed lines, and 95th percentiles are shown in dotted lines. The areas shaded in yellow correspond to the initial dose, and the areas shaded in red correspond to the subsequent three doses. Q6h, every 6 hours; Q12h, every 12 hours; Q48h, every 48 hours; S, scenario.

Simulated concentration–time profiles showed that protein C activity rapidly increased over time in all scenarios and steady state was reached approximately 48 to 60 hours after the initial dose ( Fig. 2 and Supplementary Fig. S3 ). In the three-stage dosing scenarios, the maintenance doses governed the steady-state protein C concentration achieved, while the initial dose and three subsequent doses determined initial protein C activity and how quickly the steady state was reached. A one-stage constant dose of 60 IU/kg Q12h (scenario 10) resulted in a concentration at steady state that fell between all other three-stage dosing scenarios, which had either a higher (120 IU/kg) or lower (45 IU/kg) maintenance dose, following the order of maintenance dose levels, despite the initial doses in these scenarios being 60 or 120 IU/kg.

After the first dose, *C*_max_ >100 IU/dL was predicted to be reached in a small proportion of patients (5.8–9.1%) across the three-stage dosing scenarios and in 4.8 to 44.8% of patients across the one-stage dosing scenarios (20.4–44.8% of patients at the higher one-stage dosing scenarios 11–14 [200–350 IU/kg]). *C*_trough_ >25 IU/dL was predicted to be achieved in 49.4 to 73.3% of patients with three-stage dosing, and in 43.6 to 89.0% of patients with one-stage dosing after the first dose ( Table 4 ). At the higher one-stage dosing scenarios 11 to 14, 76.6 to 89.0% of patients were predicted to achieve the target *C*_trough_ after the first dose.

**Table 4:** Percentage of patients who met target *C*_max_ and *C*_trough_ a in simulated clinical dosing scenarios after the first dose (0–12 hours) and at steady state (264–276 hours) following administration of subcutaneous protein C concentrate

Scenario	Cycle (dose in IU/kg)	*C*_max_ median (5th, 95th)	Patients with *C*_max_ >100 IU/dL, %	C _trough_ median (5th, 95th)	Patients with *C*_trough_ >25 IU/dL, %
Three-stage dosing					
S1	Post-first dose (60)	24.52 (7.67, 104.04)	5.8	24.51 (7.62, 104)	49.4
	Steady state (45)	66.64 (22.1, 163.22)	24.4	64.17 (20.63, 160.13)	91.4
S2	Post-first dose (60)	24.52 (7.67, 104.04)	5.8	24.51 (7.62, 104)	49.4
	Steady state (120)	130.79 (45.11, 297.65)	68.3	124.38 (41.98, 287.32)	99.2
S3	Post-first dose (60)	24.52 (7.67, 104.04)	5.8	24.51 (7.62, 104)	49.4
	Steady state (45)	66.67 (22.1, 163.27)	24.4	64.22 (20.63, 160.13)	91.4
S4	Post-first dose (60)	24.52 (7.67, 104.04)	5.8	24.51 (7.62, 104)	49.4
	Steady state (120)	130.79 (45.11, 297.87)	68.3	124.5 (41.98, 287.32)	99.2
S5	Post-first dose (120)	39.68 (12.62, 116.07)	9.1	39.64 (12.61, 116.03)	73.3
	Steady state (45)	66.66 (22.1, 163.27)	24.4	64.2 (20.63, 160.13)	91.4
S6	Post-first dose (120)	39.68 (12.62, 116.07)	9.1	39.64 (12.61, 116.03)	73.3
	Steady state (120)	130.79 (45.11, 297.79)	68.3	124.46 (41.98, 287.32)	99.2
S7	Post-first dose (120)	39.68 (12.62, 116.07)	9.1	39.64 (12.61, 116.03)	73.3
	Steady state (45)	66.67 (22.1, 163.27)	24.4	64.23 (20.63, 160.13)	91.4
S8	Post-first dose (120)	39.68 (12.62, 116.07)	9.1	39.64 (12.61, 116.03)	73.3
	Steady state (120)	130.8 (45.11, 298.01)	68.3	124.59 (41.98, 287.32)	99.2
One-stage dosing					
S9	Post-first dose (50 Q12h)	20.76 (7.12, 99.1)	4.8	20.75 (7.03, 99.08)	43.6
	Steady state (50 Q12h)	72.41 (23.89, 162.46)	28.4	69.81 (22.52, 159.72)	93.4
S10	Post-first dose (60 Q12h)	24.52 (7.67, 104.04)	5.8	24.51 (7.62, 104)	49.4
	Steady state (60 Q12h)	81.3 (27.09, 185.72)	35.4	78.09 (25.77, 181.58)	95.3
S11	Post-first dose (200 Q48h)	63.44 (21.9, 146.21)	20.4	44.39 (12.8, 132.07)	76.6
	Steady state (200 Q48h)	85.17 (29.41, 182.97)	38.0	53.53 (15.15, 142.42)	83.2
S12	Post-first dose (250 Q48h)	73.46 (25.45, 168.21)	29.5	50.35 (14.44, 141.29)	82.1
	Steady state (250 Q48h)	98.63 (34.91, 214.01)	48.6	60.05 (16.75, 155.01)	87.2
S13	Post-first dose (300 Q48h)	83.65 (29.58, 184.95)	36.8	56.49 (16.51, 147.51)	85.7
	Steady state (300 Q48h)	111.7 (40.24, 240.83)	58.0	66.95 (19.12, 167.27)	90.0
S14	Post-first dose (350 Q48h)	93.99 (33.22, 202.05)	44.8	62.28 (18.58, 156.27)	89.0
	Steady state (350 Q48h)	125.06 (44.89, 272.17)	66.2	73.78 (20.97, 182.52)	92.3

Abbreviations: *C*_max_ , maximum plasma concentration; *C*_trough_ , trough plasma concentration; Q12h; every 12 hours; Q48h, every 48 hours; S, scenario; SC, subcutaneous.

Each scenario is based on 2,500 simulated patients.

a The U.S. prescribing information for protein C concentrate recommends *C*_max_ >100 IU/dL and *C*_trough_ >25 IU/dL. 6

At steady state, which is representative of the long-term prophylactic treatment situation, the lowest maintenance dose of 45 IU/kg (scenarios 1, 3, 5, and 7) was predicted to result in 24.4% of patients attaining *C*_max_ >100 IU/dL. This increased to 68.3% of patients at the highest maintenance dose of 120 IU/kg (scenarios 2, 4, 6, and 8). For the one-stage dosing scenarios, 28.4 to 66.2% of patients were predicted to reach *C*_max_ >100 IU/dL at steady state. Over 90% of patients were predicted to attain *C*_trough_ >25 IU/dL at steady state with three-stage dosing scenarios 1 to 8, and similar proportions of patients (83–95%) were predicted to attain *C*_trough_ >25 IU/dL with the one-stage dosing scenarios ( Table 4 ).

**Fig. 3:**
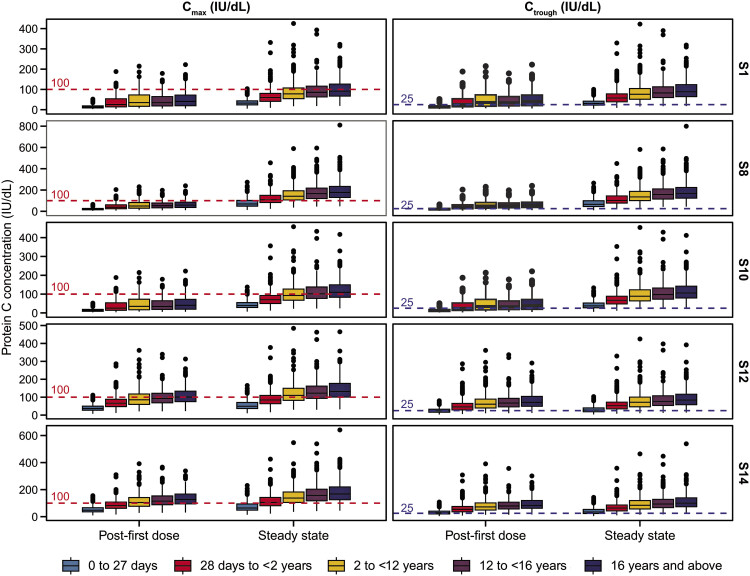
Comparison of *C*_max_ and *C*_trough_ by age group, derived from simulations of three-stage dosing at the lowest dose (S1) and highest dose (S8), and one-stage (S10, S12, and S14) dosing of subcutaneous administration of protein C concentrate. In scenario S10, patients received a constant dosage of 60 IU/kg protein C concentrate every 12 hours. Horizontal dashed lines indicate target thresholds of *C*_max_ (100 IU/dL) and *C*_trough_ (25 IU/dL). *C*_max_ , maximum plasma concentration; *C*_trough_ , plasma trough concentration; S, scenario.

When exposure parameters were stratified by age, protein C activity levels tended to increase with age, being lowest in patients <2 years of age and generally similar among the older age groups ( Fig. 3 ).

## Discussion

The potential benefits of SC administration of protein C concentrate over IV administration have been widely reported in clinical practice, including the avoidance of central venous access problems in younger children with SCPCD, as well as reduced frequency of injections. 5 18 Despite this, there are limited PK data to guide dosing via this route, which has been mostly empirical. Our study robustly characterized SC protein C concentrate PK by extending a PopPK model of IV administration 22 and utilizing SC data from the published literature. The PK characteristics of SC protein C concentrate were well-described by this model, enabling it to be used for simulations of protein C activity PK profiles for a wide range of dosing regimens of SC protein C concentrate. The findings provide important insights into the key PK characteristics of protein C activity following SC dosing, as well as comparative data to support SC treatment of severe protein C deficiency with protein C concentrate.

A trough protein C activity of ≥25 IU/dL prevents the recurrence of purpura fulminans and DIC, 9 15 and it is the maintenance trough level recommended in the product label and SmPC. 6 7 In the present analysis, simulations predicted that for all scenarios with maintenance SC dosing Q12h (three-stage scenarios 1–8 and one-stage scenarios 9 and 10), ≥91% of patients would achieve the target *C*_trough_ of >25 IU/dL at steady state. This implies that all of these dosing regimens can be effective in maintaining the target trough levels for long-term prophylactic treatment and are comparable to the predicted ≥86% of patients receiving IV protein C concentrate 22 under the same dosing regimens. However, for the initial treatment period, the trough levels were mostly below the target with the three-stage dosing regimen, suggesting that higher initial doses may be required if SC administration is used in an acute setting.

Higher and less frequent dosing could be more convenient for subpopulations of patients (e.g., neonates) and reduce patient burden. Continuous SC infusion of protein C concentrate at doses up to 350 IU/kg for Q48h has been reported previously. As noted in the EMA SmPC, SC infusion of 250–350 IU/kg protein C concentrate can produce therapeutic plasma levels. 7 In case reports of SCPCD, the calculated half-life of protein C concentrate has been reported as 16 hours for SC infusion. 10 13 Simulations were conducted of higher, less frequent dosing regimens (200–350 IU/kg Q48h, scenarios 11–14). The simulations predicted that >77% of patients would reach the target *C*_trough_ of >25 IU/dL after the first dose, indicating that these higher, less frequent doses can rapidly provide therapeutic protein C activity levels.

Higher protein C activity levels for SC administration were predicted for older age groups, owing to greater endogenous protein C production and smaller volume of distribution per kilogram of body weight. However, the difference is more pronounced for the youngest group (<2 years), which correlates with higher clearance of protein C activity in the neonates and infants, and may be explained by the significant physiological differences in this age group.

The IV 22 and SC models ( Supplementary Fig. S1 ) included both the endogenous production of protein C and its clearance (representing removal from the circulation); both processes are independent of the route of administration. The effect of disease type (SCPCD vs. SAPCD) was assessed as a covariate, and both the rate of endogenous production and the clearance of protein C were estimated to be lower in patients with SCPCD than SAPCD, with a more marked difference in the endogenous production rate (96.7% lower in patients with SCPCD). 22 These results are not expected to differ by route of administration.

The simulations predicted that 6 to 9% of patients receiving three-stage dosing (scenarios 1–8), 5 to 6% of patients receiving one-stage dosing of 50 or 60 IU/dL (scenarios 9 and 10), and 20 to 45% of patients receiving one-stage dosing of 200 to 350 IU/kg Q48h (scenarios 11–14) would achieve *C*_max_ >100 IU/dL after the first dose. The PopPK IV model has predicted higher proportions of patients (15–76%) to achieve this target after the first IV dose (60–120 IU/kg). 22 This is consistent with SC dosing typically having a slow absorption rate from the SC extracellular matrix and lower bioavailability, whereas IV dosing usually results in an immediate *C*_max_ with 100% bioavailability. 27 These results suggest that, when feasible, IV dosing of protein C concentrate is preferable to SC dosing in acute situations where rapid replacement of plasma protein C activity is needed. However, when IV access is not possible, SC doses of 200 to 350 IU/kg Q48h can be considered. In addition, SC is potentially a more convenient, less burdensome option for short- or long-term prophylactic treatment. Direct comparison of IV (scenario 8 in Li et al 22 ) and SC (scenario 14 in the present analysis) dosing regimens (matched with respect to total administered dose over an equivalent interval) is shown in Supplementary Fig. S4 .

With the SC route of administration, adverse effects appear to be limited to fibrosis, hematoma, and infection at the injection site. 5 Some authors have stated that supraphysiological activity levels of protein C concentrate should be avoided, and an estimated *C*_max_ should not exceed 150 IU/dL. 4 5 In the case reports included in the present study, no activity values >150 IU/dL were observed, and no safety concerns have been reported with SC protein C concentrate dosages up to 350 IU/kg. 10 21 26 In the present analysis, the estimated geometric mean of *C*_max_ at steady state did not exceed 125 IU/dL with SC dosing with any of the simulated dosing regimens.

Limitations of this study include limited PK data on SC administration, largely from case reports in the literature, which meant that assumptions for modeling were required. While leveraging the PopPK IV model enabled estimation of absorption model parameters for SC administration, no covariate effects or IIV could be estimated for the SC absorption parameters. Assuming that the route of administration affects only the absorption of protein C concentrate, and not its distribution or elimination, other PK parameters were fixed to the final parameter estimates in the PopPK model of protein C administered IV.

Despite these limitations, the PopPK model was robust and performed according to established standards, especially for an ultrarare disease population. Model-based simulations provided, for the first time, comprehensive insights into protein C PK at the patient population level, which are crucial for clinicians to optimize SC dosing.

## Conclusion

The PK characteristics of SC administered protein C concentrate were well-described by the PopPK model. The first and subsequent doses determine the initial levels of protein C activity and affect how fast a steady state of SC protein C concentrate can be reached, but steady-state exposure is driven by the maintenance dose levels and frequency. Model-based simulations support the use of SC dosing regimens in prophylactic settings to achieve target protein C levels of 25% with and without a loading dose. In acute settings, IV administration may still be the preferred dosing method to quickly reach the target of 100% protein C activity, although high SC doses can be considered if required. These findings suggest that SC administration of protein C concentrate has potential in the treatment of severe protein C deficiency when patients' convenience and reducing burden are important. Future clinical real-world research can provide further evidence on the effectiveness and safety of using SC protein C concentrate for the treatment of severe protein C deficiency.

## References

[1] Goldenberg N A Manco-Johnson M J Protein C deficiency Haemophilia 2008 14 06 1214 1221 19141162 10.1111/j.1365-2516.2008.01838.x

[2] Gupta A Patibandla S Protein C DeficiencyStatPearls [Internet]. StatPearls Publishing.2023. Accessed February 18, 2025 at:https://www.ncbi.nlm.nih.gov/books/NBK542222/

[3] Knoebl P N Severe congenital protein C deficiency: the use of protein C concentrates (human) as replacement therapy for life-threatening blood-clotting complications Biologics 2008 2 02 285 296 19707361 10.2147/btt.s1954 PMC2721356

[4] Manco-Johnson M J Bomgaars L Palascak J Efficacy and safety of protein C concentrate to treat purpura fulminans and thromboembolic events in severe congenital protein C deficiency Thromb Haemost 2016 116 01 58 68 27052576 10.1160/TH15-10-0786

[5] Minford A Brandão L R Othman M Diagnosis and management of severe congenital protein C deficiency (SCPCD): Communication from the SSC of the ISTH J Thromb Haemost 2022 20 07 1735 1743 35570324 10.1111/jth.15732

[6] Baxalta US. Inc.CEPROTIN. Prescribing information 2023. Accessed February 18, 2025 at:https://www.shirecontent.com/PI/PDFs/CEPROTINHCP_USA_ENG.pdf

[7] Takeda Manufacturing Austria AG. CEPROTIN.Summary of Product Characteristics 2023. Accessed February 18, 2025 at:https://www.ema.europa.eu/en/documents/product-information/ceprotin-epar-product-information_en.pdf

[8] Boey J P Jolley A Nicholls C Novel protein C gene mutation in a compound heterozygote resulting in catastrophic thrombosis in early adulthood: diagnosis and long-term treatment with subcutaneous protein C concentrate Br J Haematol 2016 172 05 811 813 26103879 10.1111/bjh.13538

[9] de Kort E H Vrancken S L van Heijst A F Binkhorst M Cuppen M P Brons P P Long-term subcutaneous protein C replacement in neonatal severe protein C deficiency Pediatrics 2011 127 05 e1338 e1342 21482600 10.1542/peds.2009-2913

[10] Minford A Behnisch W Brons P Subcutaneous protein C concentrate in the management of severe protein C deficiency–experience from 12 centres Br J Haematol 2014 164 03 414 421 24422725 10.1111/bjh.12640

[11] Olivieri M Kurnik K Engelsberger I Bidlingmaier C Management of subcutaneous protein C substitution in a child with severe protein C deficiency Hamostaseologie 2009 29 01 S103 S104

[12] Piccini B Capirchio L Lenzi L Continuous subcutaneous infusion of protein C concentrate using an insulin pump in a newborn with congenital protein C deficiency Blood Coagul Fibrinolysis 2014 25 05 522 526 24509341 10.1097/MBC.0000000000000079

[13] Sanz-Rodriguez C Gil-Fernández J J Zapater P Long-term management of homozygous protein C deficiency: replacement therapy with subcutaneous purified protein C concentrate Thromb Haemost 1999 81 06 887 890 10404762

[14] Human protein C: new preparations. Effective replacement therapy for some clotting disorders Prescrire Int 2003 12 63 11 13 12602374

[15] Dreyfus M Masterson M David M Replacement therapy with a monoclonal antibody purified protein C concentrate in newborns with severe congenital protein C deficiency Semin Thromb Hemost 1995 21 04 371 381 8747700 10.1055/s-2007-1000658

[16] Mathias M Khair K Burgess C Liesner R Subcutaneous administration of protein C concentrate Pediatr Hematol Oncol 2004 21 06 549 554 10.1080/08880010490477365 15552819

[17] Shah R Ferreira P Karmali S Le D Severe congenital protein C deficiency: practical aspects of management Pediatr Blood Cancer 2016 63 08 1488 1490 27138381 10.1002/pbc.25997

[18] Chaubal M V Dedík L Durisová M Bruley D F Modeling behavior of protein C during and after subcutaneous administration Adv Exp Med Biol 2005 566 389 395 16594177 10.1007/0-387-26206-7_51

[19] Wang M Hertfelder H-J Swallow E Real-world treatment of patients with severe congenital protein C deficiency with protein C concentrate: A physician survey Thromb Update 2024 14 100159

[20] US Food and Drug Administration.Guidance for Industry: Population Pharmacokinetics 2022. Accessed February 18, 2025 at:https://www.fda.gov/media/128793/download

[21] Pöschl J Behnisch W Beedgen B Kuss N Case report: successful long-term management of a low-birth weight preterm infant with compound heterozygous protein C deficiency with subcutaneous protein C concentrate up to adolescence Front Pediatr 2021 9 591052 34650936 10.3389/fped.2021.591052 PMC8506145

[22] Li Z Sorribes I C Schneider J Taylor A Evaluation of pharmacokinetics of intravenous protein C concentrate in protein C deficiency: implications for treatment initiation and maintenance Res Pract Thromb Haemost 2025 9 03 102859 40492255 10.1016/j.rpth.2025.102859 PMC12146541

[23] European Medicines Agency (EMA), Committee for Medicinal Products for Human Use (CHMP).Guideline on reporting the results of population pharmacokinetic analyses, Doc. RefCHMP/EWP/185990/06, London, June 21,2007. Accessed February 18, 2025 at:https://www.ema.europa.eu/en/documents/scientific-guideline/guideline-reporting-results-population-pharmacokinetic-analyses_en.pdf

[24] Centers for Disease Control, National Center for Health Statistics.Third National Health and Nutrition Examination Survey (NHANES III) 1997. Accessed February 18, 2025 at:https://wwwn.cdc.gov/nchs/nhanes/nhanes3/default.aspx

[25] Willmann S Lippert J Sevestre M Solodenko J Fois F Schmitt W PK-Sim®: a physiologically based pharmacokinetic ‘whole-body’ model BIOSILICO 2003 1 121 124

[26] Minford A M Parapia L A Stainforth C Lee D Treatment of homozygous protein C deficiency with subcutaneous protein C concentrate Br J Haematol 1996 93 01 215 216 8611462 10.1046/j.1365-2141.1996.4691021.x

[27] Bittner B Richter W Schmidt J Subcutaneous administration of biotherapeutics: an overview of current challenges and opportunities BioDrugs 2018 32 05 425 440 30043229 10.1007/s40259-018-0295-0 PMC6182494

