# Supplementary material for: Pharmacokinetic Evidence Supporting Subcutaneous Use of Protein C Concentrate in Patients with Protein C Deficiency
Source: TH Open. 2025 Nov 11;9:a27315372. doi: 10.1055/a-2731-5372 (PMC12694723; doi:10.1055/a-2731-5372)
Supplement: Supplementary file 1 — Supplementary Material [file 10-1055-a-2731-5372_27439132.pdf]

<u>**SUPPLEMENTARY MATERIAL**</u>

Supplementary Methods

PopPK model dataset

The literature summaries did not include all the information necessary for the analysis, so several data imputations were made. In cases where indication information was missing, indication was imputed to congenital. The data missing for each source were different and handled on an individual basis as follows:

1. Boey JP, et al. 2016:[1] Duration of SC administration was assumed to be 30 minutes, and the baseline value was assumed to be half of the lower limit of quantification (2.5 IU/dL).
2. de Kort EH, et al. 2011:[2] Samples were assumed to have been collected 1 h before the administered dose.
3. Minford A, et al. 2014:[3] Predose was assumed to be 1 h before the dose, post-dose was assumed to be 11 h after the dose, and trough was assumed to be 1 h before the next dose. Body weight was imputed based on age per US Centers for Disease Control growth charts [4]. When dose was reported as “30-50,” a dose of 40 IU/kg was chosen. Sex was missing in all subjects and imputed to “Male.” Symptomatic status was also missing and imputed to “symptomatic.”
4. Olivieri M, et al. 2009:[5] Duration was assumed to be 30 minutes.
5. Piccini B, et al. 2014:[6] Age was considered to be 1 day old.
6. Sanz-Rodriguez C, et al. 1999:[7] Age was considered to be 1 day old and body weight was imputed to 2.7 kg.

PopPK model development

The PopPK model for SC protein C concentrate administration was based on a previously developed model for IV protein C concentrate.[8] The model was a one-compartment linear model with first-order elimination, endogenous protein C production, and body weight–based

TH Open | DOI 10.1055/a-2731-5372 | © The Author(s).

allometric scaling of clearance and volume of distribution. PK data for SC protein C concentrate from the literature were combined with the analysis dataset previously used to develop the PopPK model for IV protein C concentrate in an exploratory data analysis. As administration of a drug by any route other than the systemic (IV) route introduces an absorption step, an absorption compartment was added to the structural model to accommodate SC data. Given sparsity of SC data, instead of estimating all parameters at once using SC and IV data, all parameters estimated in the IV model were fixed. Bioavailability, duration of administration, and absorption rate constant were estimated using only the SC data.

Owing to limited individual-level data in the literature, no IIV was incorporated into the SC absorption parameters. As information on covariates was limited in the literature, no covariate analysis was performed after the addition of SC data into the model.

TH Open | DOI 10.1055/a-2731-5372 | © The Author(s).

Supplementary Figures

Supplementary Figure S1. Structural schematic for the final PopPK model to describe plasma concentrations of protein C following SC administration of protein C concentrate in patients with SCPCD. CL, clearance; D, duration of administration; KA, absorption rate; PopPK, population pharmacokinetic; SC, subcutaneous; SCPCD, severe congenital protein C deficiency; V_d, volume of distribution.

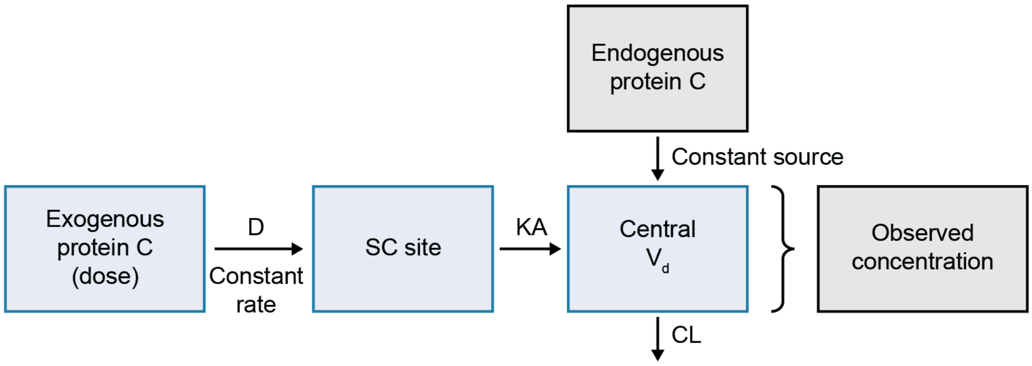

TH Open | DOI 10.1055/a-2731-5372 | © The Author(s).

Thieme

Supplementary Figure S2. Standard goodness-of-fit plots for the final subcutaneous protein C PopPK model. CWRES, conditional weighted residuals; PopPK, population pharmacokinetic.

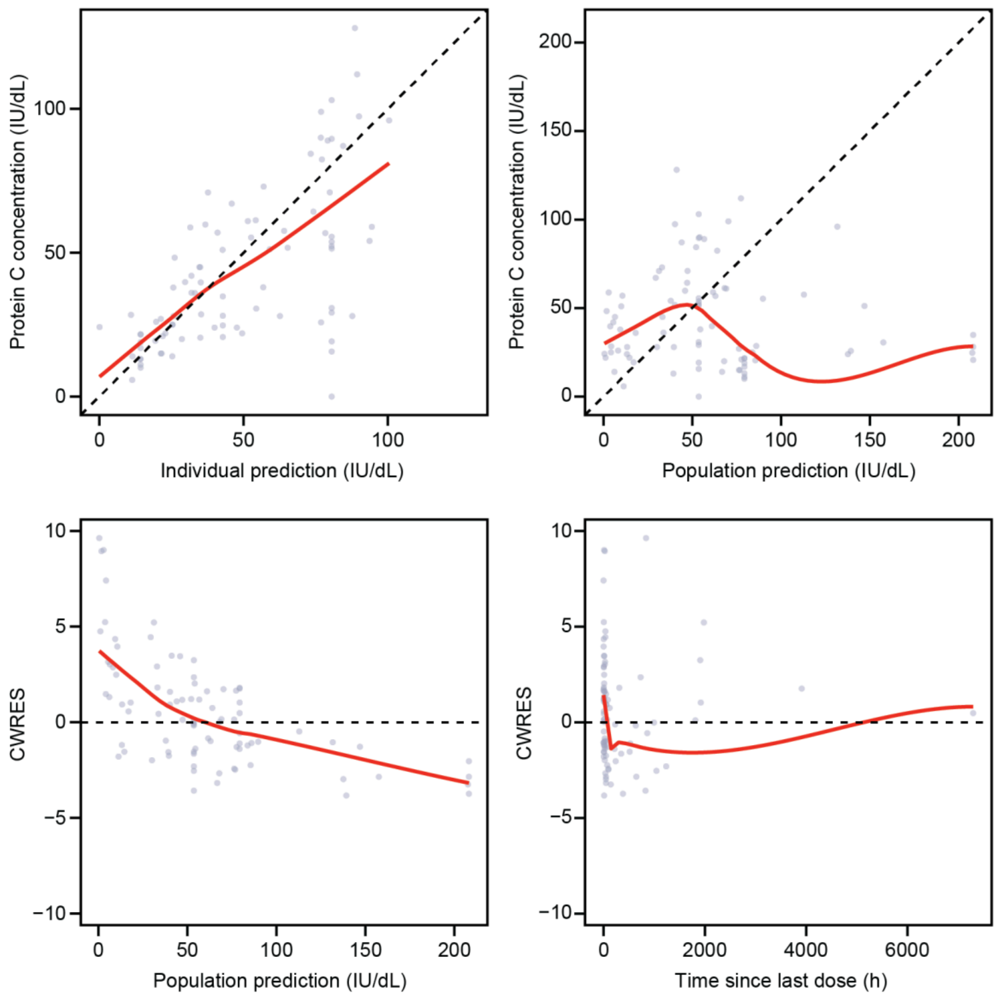

TH Open | DOI 10.1055/a-2731-5372 | © The Author(s).

Supplementary Figure S3. Simulated protein C concentrate concentration–time profiles showing representative 3-stage and 1-stage dosing scenarios (S2 to S7, S9, S11, and S13). Details of the dosing regimen in each scenario are shown in Table 1. Median values are shown in solid lines, 5th percentiles are shown in dashed lines, and 95th percentiles are shown in dotted lines. Area shaded in yellow corresponds to the initial dose and area shaded in red corresponds to the subsequent 3 doses. S, scenario.

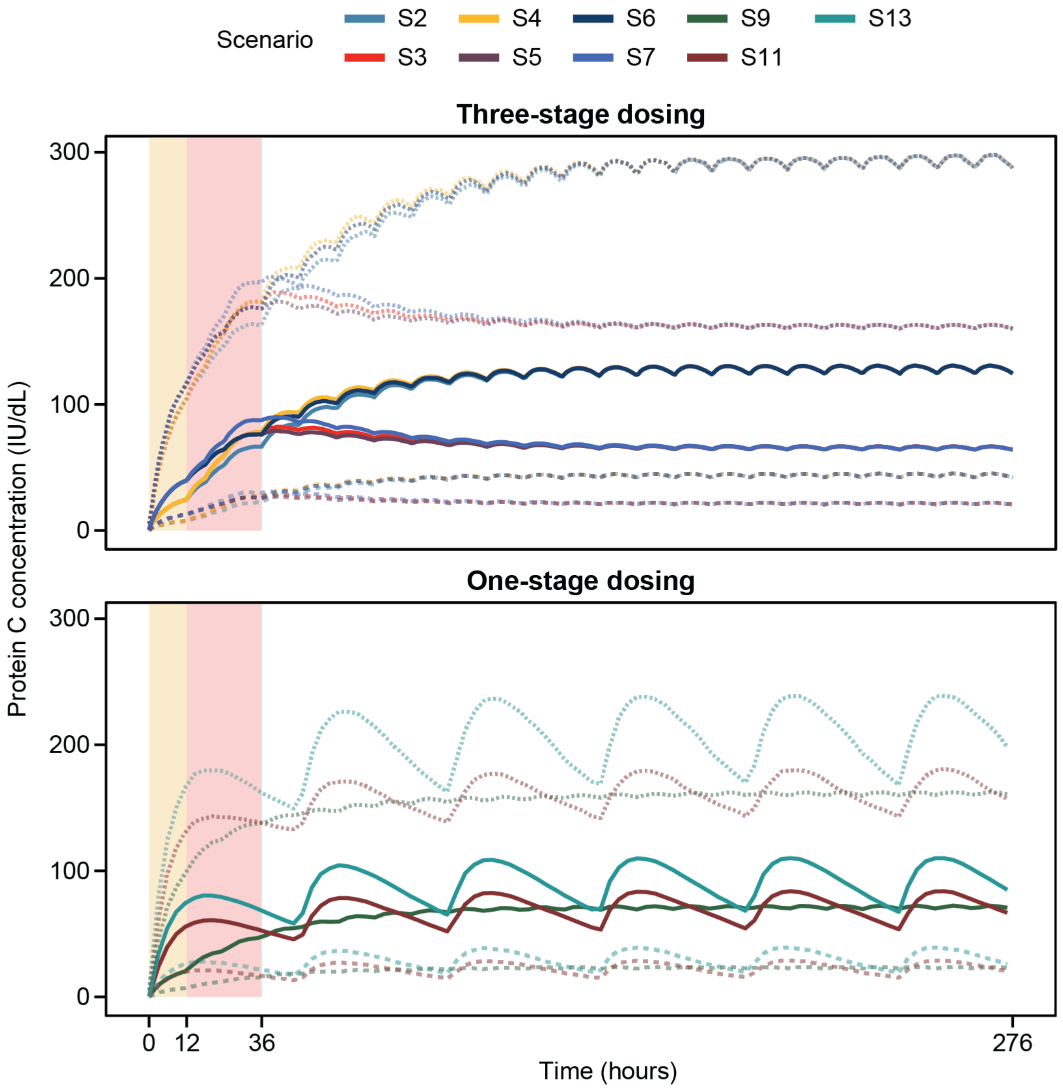

TH Open | DOI 10.1055/a-2731-5372 | © The Author(s).

Supplementary Figure S4. Simulated protein C concentrate concentration–time profiles showing representative 3-stage IV (S8 in Li Z, et al.[8]) and 1-stage SC (S14 in the present analysis) dosing scenarios. Median values are shown in solid lines, 5th percentiles are shown in dashed lines, and 95th percentiles are shown in dotted lines. Area shaded in yellow corresponds to the initial dose (3-stage and 1-stage dosing) and area shaded in red corresponds to the subsequent 3 doses (3-stage dosing only). IV, intravenous; Q6h, every 6 hours; Q12h, every 12 hours; Q48h, every 48 hours; S, scenario; SC, subcutaneous.

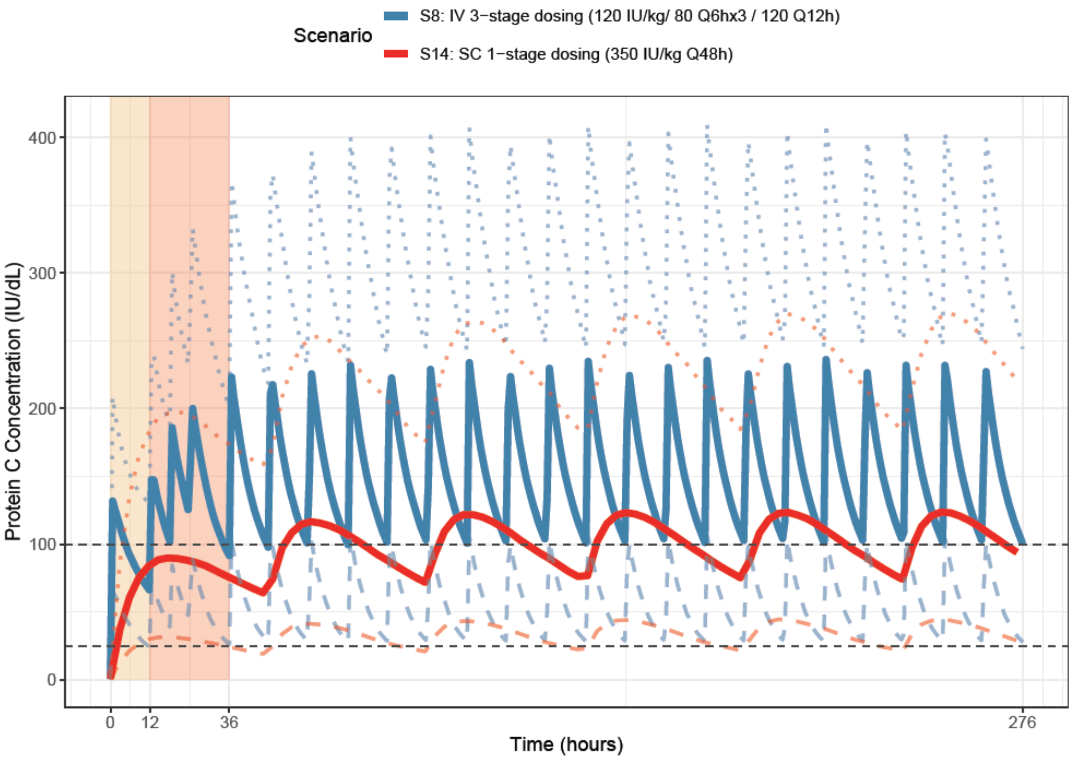

TH Open | DOI 10.1055/a-2731-5372 | © The Author(s).

References

1. Boey JP, Jolley A, Nicholls C, et al. Novel protein C gene mutation in a compound heterozygote resulting in catastrophic thrombosis in early adulthood: diagnosis and long-term treatment with subcutaneous protein C concentrate. Br J Haematol 2016;172(5):811–813
2. de Kort EH, Vrancken SL, van Heijst AF, Binkhorst M, Cuppen MP, Brons PP. Long-term subcutaneous protein C replacement in neonatal severe protein C deficiency. Pediatrics 2011;127(5):e1338–1342
3. Minford A, Behnisch W, Brons P, et al. Subcutaneous protein C concentrate in the management of severe protein C deficiency – experience from 12 centres. Br J Haematol 2014;164(3):414–421
4. Centers for Disease Control, National Center for Health Statistics. Third National Health and Nutrition Examination Survey (NHANES III) 1997. Accessedat: https://wwwn.cdc.gov/nchs/nhanes/nhanes3/default.aspx
5. Olivieri M, Kurnik K, Engelsberger I, Bidlingmaier C. Management of subcutaneous protein C substitution in a child with severe protein C deficiency. Hamostaseologie 2009;29 (Suppl 1):S103–104
6. Piccini B, Capirchio L, Lenzi L, et al. Continuous subcutaneous infusion of protein C concentrate using an insulin pump in a newborn with congenital protein C deficiency. Blood Coagul Fibrinolysis 2014;25(5):522–526
7. Sanz-Rodriguez C, Gil-Fernández JJ, Zapater P, et al. Long-term management of homozygous protein C deficiency: replacement therapy with subcutaneous purified protein C concentrate. Thromb Haemost 1999;81(6):887–890
8. Li Z, Sorribes IC, Schneider J, Taylor A. Evaluation of pharmacokinetics of intravenous protein C concentrate in protein C deficiency: implications for treatment initiation and maintenance. Res Pract Thromb Haemost 2025;9(3):102859

TH Open | DOI 10.1055/a-2731-5372 | © The Author(s).
